# Superiority of the Non-Glycosylated Form over the Glycosylated Form of Irisin in the Attenuation of Adipocytic Meta-Inflammation: A Potential Factor in the Fight against Insulin Resistance

**DOI:** 10.3390/biom9090394

**Published:** 2019-08-21

**Authors:** Agnieszka Irena Mazur-Bialy

**Affiliations:** Department of Ergonomics and Exercise Physiology, Institute of Physiotherapy, Faculty of Health Science, Jagiellonian University Medical College, Grzegorzecka 20, 31-531 Krakow, Poland; agnieszka.mazur@uj.edu.pl; Tel.: +48-12-421-9351

**Keywords:** non-glycosylated irisin, glycosylated irisin, meta-inflammation, obesity, insulin resistance, metabolic syndrome, adipomyokine, physical exercise

## Abstract

Irisin is an adipomyokine that promotes the browning of white adipose tissue and exhibits protective potential against the development of insulin resistance and type 2 diabetes. In our bodies, it occurs in its glycosylated form (G-IR): its activity is still poorly understood, because the majority of studies have used its non-glycosylated counterpart (nG-IR). Glycosylation can affect protein function: therefore, the present study attempted to compare the actions of both forms of irisin toward inflammatory activation of the main component of adipose tissue. The study was carried out in a coculture of 3T3 adipocytes and RAW 264.7 macrophages maintained in the presence of nG-IR or G-IR. The impact on vitality and the expression and release of key inflammatory mediators important for insulin resistance and diabetes development were assessed. The studies showed that both forms effectively inhibited the expression and release of tumor necrosis factor (TNF)-α, interleukin (IL)-1β, IL-6, macrophage chemotactic protein (MCP)-1, high-mobility group box (HMGB1), leptin, and adiponectin. However, in the case of TNF-α, IL-1β, MCP-1, and HMGB1, the inhibition exerted by nG-IR was more prominent than that by G-IR. In addition, only nG-IR significantly inhibited macrophage migration. Here, nG-IR seemed to be the stronger inhibitor of the development of obesity-related inflammation; however, G-IR also had anti-inflammatory potential.

## 1. Introduction

Obesity is a serious civilization problem that has now reached pandemic dimensions, with nearly 1.9 million people being obese or overweight. Obesity has also resulted in a significant increase in obesity-related diseases such as type 2 diabetes, insulin resistance, cardiovascular disease, or even cancer. Up to 50 million children under the age of five could be at risk for obesity [1,2]. Moreover, studies have shown that excessive accumulation of fat in white adipose tissue (WAT) is accompanied by the development of moderate chronic inflammation [3] and underlying complications associated with obesity, and that the macrophage population resident in adipose tissue is the main source of proinflammatory factors [2,4,5]. Our previous research of an independent culture of adipocytes [6] and macrophages [7,8,9] showed that irisin directly affects the intensity of its proinflammatory activation. Given the proinflammatory basis of numerous complications accompanying obesity, meta-inflammation is observed in adipose tissue, and this underlies the development of chronic obesity-related inflammation [6]. Therefore, the search for agents that inhibit meta-inflammation has become particularly important.

Irisin is an adipomyokine that was discovered in 2012 by Boström et al. [10] as a new factor promoting thermogenesis through the transformation of white adipose tissue (WAT) into thermogenic beige adipose tissue (BAT) [3,10,11]. After a controversy related to its actual existence and the possibility of its detection in humans [12,13], irisin was finally confirmed using a mass spectrometry technique in 2015 by Jedrychowski et al. [14]. Irisin was quickly characterized as a potential valuable factor in the fight against obesity, insulin resistance, and type 2 diabetes [11,15,16,17,18]. Numerous reports have indicated a significant downregulation of the irisin level in the above-mentioned states [19,20,21], but current evidence suggests that during the course of obesity, the irisin level is elevated and a state of irisin resistance is observed [22,23]. Studies on irisin activity seem to be particularly important because of the increase in its release after physical exercise, as confirmed by numerous previous studies (for review, see Reference [24]). This may suggest the possibility of using physical exercise stimulation to modulate the irisin level and to achieve a potential therapeutic effect. Irisin is a small protein with a length of only 112 amino acids and results from the proteolytic cleavage of the fibronectin type III domain containing five proteins (FNDC5) [10], which has two *N*-glycosylation sites [25]. Glycosylation affects the properties of numerous proteins and can affect the modulation of many biological processes [26]. For example, irisin glycosylation affects its half-life by stabilizing its structure [27,28]. Moreover, some studies have indicated that the glycosylation of irisin affects its effectiveness [29]. For example, Gannon et al. [29] showed that irisin glycosylation weakens anticancer activity, which he observed after the administration of a non-glycosylated form. Despite numerous studies on irisin function [6,7,8,9], the mechanism of action of the glycosylated form is still poorly understood. This lack of understanding is caused by the fact that most research is performed on the non-glycosylated form of irisin (nG-IR), despite the fact that, according to Gannon et al. [29], differences in the action of these two forms have been observed.

In connection with the above, the aim of the research envisaged in this study was to compare the biological activity of the two different forms of irisin in the case of proinflammatory activation of adipocyte–macrophage cocultures. Given that the non-glycosylated form of irisin inhibits the proinflammatory activity of adipocytes [6] and macrophages [7,8,9], we verified whether its glycosylation changes the nature of its impact on the equivalent of obesity-related meta-inflammation. In particular, we evaluated the effect of irisin glycosylation on the expression and release of key proinflammatory cytokines, including tumor necrosis factor (TNF) α, interleukin (IL)-1β, IL-6, macrophage chemotactic protein (MCP)-1, high-mobility group box (HMGB)1, and adiponectin and leptin adipokines. We also studied the effectiveness of irisin forms in inhibiting the migration activity of macrophages to supernatants from cocultures as a counterpart to the macrophage influx into adipose tissue.

## 2. Materials and Methods 

### 2.1. Chemicals and Materials 

Dulbecco’s Modified Eagle Medium (DMEM), antibiotics (streptomycin and penicillin), and fetal bovine serum (FBS) were purchased from PAA (Pasching, Austria). A CellTiter kit was purchased from Promega (Madison, WI, USA). Lipopolysaccharide (LPS) was obtained from Sigma Aldrich (St. Louis, MO, USA). The RNeasy Plus Mini Kit (74134) for RNA isolation was purchased from Qiagen (Hilden, Germany). The High Capacity RNA-to-cDNA Kit (4387406), TaqMan Gene Expression Master Mix (4369026), and oligo(dT) primer (TNF-α Assay ID: Mm00443260_g1; IL-6 Assay ID: Mn00446190_m1; MCP-1 Assay ID: Mm00441242_m1; HMGB1 Assay ID: Mm00805422_m1; adiponectin Assay ID: Mm00456425_m1; leptin Assay ID: Mm00434759_m1; GAPDH Assay ID: Mm99999915_g1) were obtained from Applied Biosystems (Foster City, CA, USA). Mouse adiponectin enzyme-linked immunosorbent assay (ELISA) kits were purchased from NOVEX (ThermoFisher; Foster City, CA, USA). ELISA kits for TNFα, IL-1β, IL-6, HMGB1, and leptin were obtained from IBL International (Hamburg, Germany). ELISA kits for mouse MCP-1 were purchased from R&D BioVendor (Heidelberg, Germany).

### 2.2. Cell Coculture and Experimental Design 

The study was conducted on a Mycoplasma-free coculture of 3T3 L1 murine pre-adipocytes differentiated into mature adipocytes and a murine RAW 264.7 macrophage cell line. The RAW 264.7 cell line was purchased from the European Type Culture Collection (ETCC, Sigma Aldrich), and the 3T3 L1 pre-adipocytes were kindly provided by Professor Alicja Józkowicz from the Department of Medical Biology, Jagiellonian University, Kraków. The differentiation of pre-adipocytes into adipocytes was performed according to a protocol described previously [6]. The efficiencies of adipocyte differentiation and lipid droplet deposition were assessed using Oil-Red-O staining according to the manufacturer’s instructions. 

For the coculture study, cells were maintained under standard conditions (37 °C, 5% CO_2_) in a DMEM medium supplemented with 10% fetal bovine serum (FBS), 1% antibiotics, and 1 µg/mL of insulin. Fully differentiated adipocytes were cultured in the lower parts of 12-well plates, while the RAW 264.7 cells were cultured in plate inserts (0.4-µm pore size). Cells were cultured in the presence of glycosylated (G-IR) or non-glycosylated (nG-IR) forms of irisin (0–100 nM) for 24 h. Then, in order to cause induction of a state of mild inflammation, cocultures were stimulated with lipopolysaccharide (LPS; 100 ng/mL; *Escherichia coli*, serotype 0111: B4) for the next 6 (gene expression studies) or 24 h (protein release studies). Fresh cells were used for cytometric analyses and RNA isolation. Supernatants and cell pellets were frozen (−60 °C) for future quantification of protein levels. 

### 2.3. Cell Activity and Viability 

Overall cell viability and activity were determined after 24 h of coculture using a commercial CellTiter kit according to the manufacturer’s instructions on a spectrophotometer (Expert Plus, ASYS/Hitech). The overall cell activity was assessed after 2 h of culture in an irisin-enriched environment, while proliferation was assessed after 24 h of culture.

### 2.4. Quantitative Real-Time PCR Assay 

Total cell RNA from adipocytes and macrophages was extracted using an RNeasy Plus Mini Kit with the elimination of genomic DNA according to the manufacturer’s instructions. The concentration and quality of RNA were measured using a NanoDrop 2000 spectrophotometer (Thermo Scientific, Waltham, MA, USA). Reverse transcription for cDNA synthesis was performed using a High Capacity cDNA Reverse Transcription Kit in Thermoblock Thermostat Plus (Eppendorf) according to the manufacturer’s protocol. Real-time PCR gene expression analysis was performed using Taq Man oligo(dT) primer (for TNF-α, IL-1β, IL-6, MCP-1, HMGB1, adiponectin, leptin) and TaqMan Gene Expression Master Mix in the StepOne Plus system (Applied Biosystems). The expressions of the analyzed genes were normalized to endogenous glyceraldehyde phosphate dehydrogenase (GAPDH) gene expression, which was selected as the housekeeping gene. The relative expression of each gene (RQ) was calculated using the 2^−∆∆Ct^ method.

### 2.5. Examination of Cytokine and Adipokine Release 

After 24 h incubation of coculture carried out with or without a LPS presence, the supernatants were collected, and the levels of cytokines and adipokines were quantified using a commercial ELISA kit. The levels of TNFα, IL-1β, IL-6, HMGB1, MCP-1, adiponectin, and leptin were determined according to the manufacturer’s instructions and measured using an Expert Plus spectrophotometer (ASYS/Hitech, Eugendorf, Austria).

### 2.6. Macrophage Migration 

The migration of RAW 264.7 macrophages toward the supernatant collected from the coculture was evaluated using a 48-well microchemotaxis chamber (Neuro Probe). The test was performed according to the manufacturer’s protocol. Briefly, the lower wells of the chamber were filled with adipocyte or coculture supernatants collected 24 h after LPS stimulation, control supernatants without LPS stimulation, *N*-Formylmethionyl-leucyl-phenylalanine (fMLP) as a positive control, or DMEM medium as a negative control. The lower wells of the chamber were covered with a polycarbonate membrane (3-µm pore size; Neuro Probe). The wells in the upper chamber were filled with 50 µL of RAW 264.7 macrophages (2 mln/mL) and incubated for 40 min in standard culture conditions. Thereafter, the membrane was washed in PBS and stained with Diff-Quick solution (Medion Diagnostic). The macrophages that migrated to the lower side of the membrane were counted in four microscopic fields in each well. The results are expressed as the mean numbers of migratory macrophages per well.

### 2.7. Statistical Analysis 

Data were tested for the normality of distribution, and differences between groups were determined using Duncan’s new multiple range test 3.1. All data are expressed as mean ± standard deviation (*X* ± SD), with the level of statistical significance (*p*) set at 0.05.

## 3. Results

The non-glycosylated form of irisin is known to modulate the proinflammatory activation of both adipocytes [6] and macrophages [7,8,9]. However, the endogenous form of irisin typical of eukaryotic organisms is the glycosylated form. Despite this, there have still been no studies comparing the effects of these two forms of irisin on the development of obesity-related inflammation. In the present study, the impact of irisin supplementation was analyzed with respect to the coculture of the above-mentioned cells: both of these exhibited inflammatory activation and participated in the development of chronic obesity-related inflammation.

### 3.1. Overall Activity and Viability of Cell Coculture

**Figure 1:**
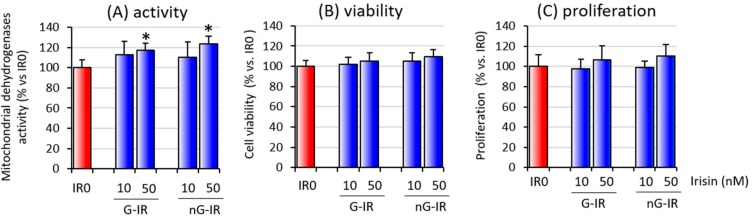
Effects of various forms of irisin (glycosylated (G-IR) or non-glycosylated (nG-IR)) on overall cell activity measured in an CellTiter test (**A**). Viability (**B**) and proliferation (**C**) of adipocyte–macrophage cocultures. Fully differentiated adipocytes (3T3 L1) and macrophages (RAW 264.7) were cultured in 12-well dishes with inserts (0.4-μm pore size) for 24 h with or without the presence of irisin (0, 10, or 50 nM; IR0, IR10, or IR50, respectively). The results are expressed as mean + standard deviation (SD) from five independent tests. Statistical significances were determined with the Duncan *t*-test. The asterisks (*) above the bars mark statistically significant differences (*p* < 0.05) calculated relative to the IR0 group.

The first stage in this research was an evaluation of the influence of the two forms of irisin (glycosylated and non-glycosylated) on the overall viability and activity of cell cocultures. The studies showed no differences between the effect induced by either form of irisin, but in both cases an increase in the overall activity of coculture cells (nG-IR50 and G-IR50), with no effect on cell viability and proliferation, was observed (Figure 1). 

### 3.2. Cytokine Production Was More Strongly Inhibited by the Non-Glycosylated Form of Irisin

**Figure 2:**
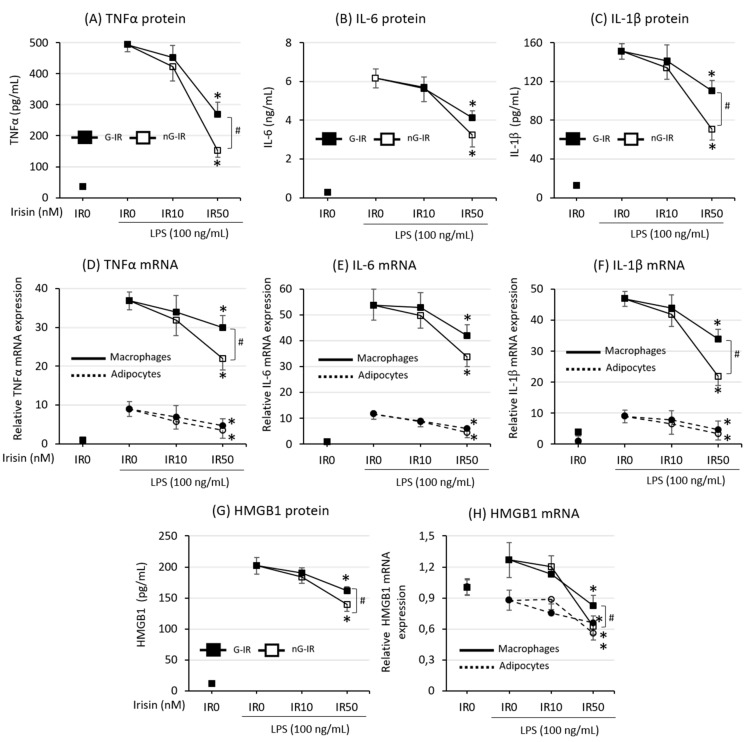
Effects of various forms of irisin (glycosylated (G-IR) or non-glycosylated (nG-IR)) on lipopolysaccharide (LPS)-induced cytokine release: tumor necrosis factor-alpha (TNF-α) (**A**), interleukin 6 (IL-6) (**B**), and interleukin 1β (IL-1β) (**C**). Cytokine mRNA expression: TNF-α (**D**), IL-6 (**E**), IL-1β (**F**), as well as high-mobility group box-1 protein (HMGB1) release (**G**) and HMGB1 mRNA expression (**H**), by adipocyte–macrophage cocultures. Fully differentiated 3T3 L1 adipocytes and RAW 264.7 macrophages were cultured in 12-well dishes with inserts (0.4-μm pore size) for 6 h (mRNA expression, RT-PCR) or 24 h (cytokine release, ELISA tests) with or without the presence of irisin (0, 10, or 50 nM) administered 2 h before LPS stimulation (100 ng/mL). The results are expressed as mean + standard deviation (SD) from five independent experiments. Statistical significances (*p* < 0.05) were determined with the Duncan *t*-test. The asterisk (*) above the bar denotes statistically significant differences in protein or mRNA level calculated relative to the IR0 group, while the hash (#) denotes statistically significant differences calculated between the G-IR and the nG-IR group.

The next stage in the investigation was an evaluation of the effect of irisin on the intensity of the production and release of key proinflammatory cytokines, such as TNF-α, IL-1β, IL-6, and HMGB1. The studies showed that irisin significantly inhibited both the production and release of proinflammatory cytokines, regardless of the presence or absence of glycosylation. However, the non-glycosylated form of irisin had a much stronger effect than its counterpart did (Figure 2; *p* < 0.05 for TNF-α, IL-1β, and HMGB1). The inhibition of cytokine production was observed in TNF-α (Figure 2A), IL-1β (Figure 2B), and IL-6 (Figure 2C), as well as in HMGB1 (Figure 2G). In each of these cases, only the concentration of 50 nM of irisin was effective. The expression of these cytokines at the mRNA level showed that the decrease in cytokine release was dependent on the inhibition of the expression of these cytokines in both macrophage and adipocyte cells (Figure 2D–F,H, respectively). Nevertheless, the level of cytokine expression in macrophages was much higher than in adipocyte cells. This indicated the coproduction of obesity-related inflammation by both cell populations that build adipose tissue.

### 3.3. Both Forms of Irisin Similarly Inhibited the Production of Adipokines

**Figure 3:**
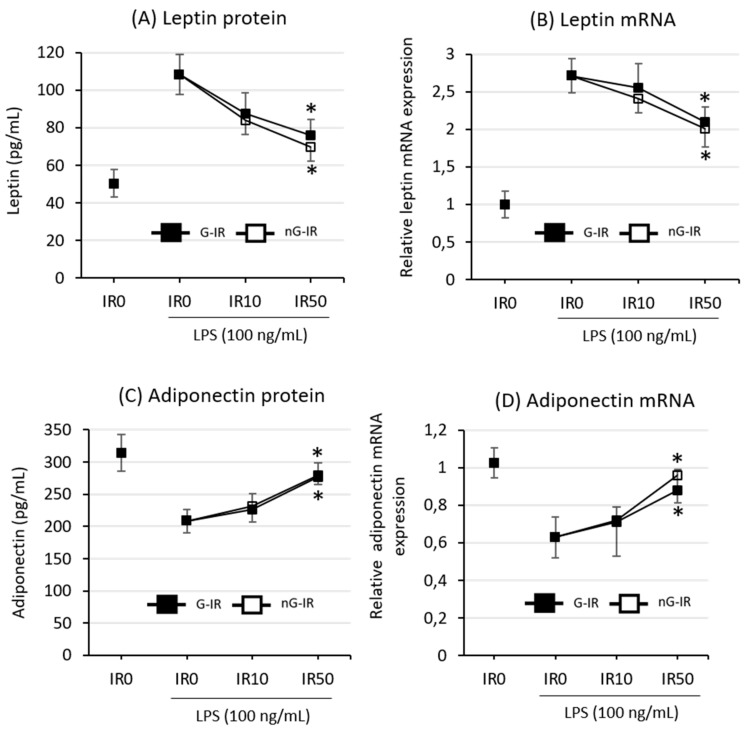
Effects of various forms of irisin (glycosylated (G-IR) or non-glycosylated (nG-IR)) on LPS-induced leptin release (**A**) or mRNA expression (**B**) and adiponectin release (**C**) or mRNA expression (**D**) by adipocyte–macrophage cocultures. Fully differentiated 3T3 L1 adipocytes and RAW 264.7 macrophages were cultured in 12-well dishes with inserts (0.4-μm pore size) for 6 h (mRNA expression, RT-PCR) or 24 h (adipokine release, ELISA tests) with or without the presence of irisin (0, 10, or 50 nM; IR0, IR10, or IR50, respectively). The results are expressed as mean + standard deviation (SD) from five independent experiments. Statistical significances (*p* < 0.05) were determined with the Duncan *t*-test. The asterisk (*) above the bar denotes statistically significant differences in protein or mRNA levels, calculated relative to the IR0 group.

An assessment of the effect of irisin treatment on the production and release of adipokines showed that the presence or absence of glycosylation did not affect the nature of irisin action. However, it was noted that irisin at 50 nM effectively inhibited both the expression (Figure 3A) and the release of leptin, referred to as a proinflammatory adipokine (Figure 3B), while it increased the expression and release of the anti-inflammatory adiponectin (Figure 3C,D, respectively). It was not observed that any of the forms of irisin had a stronger effect: their effects were comparable.

### 3.4. Non-Glycosylated Form of Irisin Inhibited Macrophage Migration

**Figure 4:**
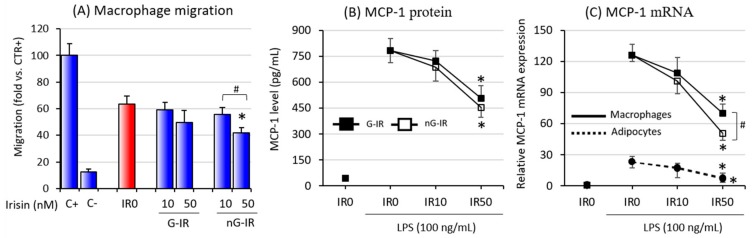
Effects of various forms of irisin (glycosylated (G-IR) or non-glycosylated (nG-IR)) on (**A**) RAW 264.7 macrophage migration to conditioned medium collected from adipocytes cultured in the presence of various concentrations of irisin (0, 10, or 50 nM) for 24 h. Normal medium was used as a negative control (C-); *N-*Formylmethionyl-leucyl-phenylalanine (fMLP) was used as a positive control (C+). Irisin effects on LPS-induced monocyte chemotactic protein 1 (MCP-1) release (**B**) and MCP-1 mRNA expression (**C**) by adipocyte–macrophage cocultures. Fully differentiated 3T3 L1 adipocytes and RAW 264.7 macrophages were cultured in 12-well dishes with inserts (0.4-μm pore size) for 6 h (mRNA expression, RT-PCR) or 24 h (MCP-1 release, ELISA tests) with or without the presence of irisin (0, 10, or 50 nM) administered 2 h before LPS stimulation (100 ng/mL). The results are expressed as mean + SD from five independent experiments. Statistical significances (*p* < 0.05) were determined with the Duncan *t*-test. The asterisk (*) above the bar denotes statistically significant differences in protein or mRNA levels calculated relative to the IR0 group, while the hash (#) denotes statistically significant differences calculated between the G-IR and the nG-IR groups.

Given that macrophage influx into adipose tissue exists as a result of chemotactic factors released by fat tissue cells and significantly contributes to the development of moderate inflammation associated with obesity, in the last stage of the study, we assessed the effects of both types of irisin on macrophage migration. The studies showed that the glycosylated form of irisin at 50 nM significantly reduced the degree of macrophage migration toward the coculture supernatant (Figure 4A). The intensity of macrophage migration to the supernatant from G-IR50 cocultures was slightly reduced, which was in line with previous studies where effective inhibition was observed only in the IR100 group [6]. The studies also showed that this phenomenon may be associated with a significant reduction in both the expression and release of the key chemokine, MCP-1, when the coculture was grown in an environment enriched with irisin at a concentration of 50 nM (Figure 4B). As the study showed, the expression of MCP-1 was significantly higher in macrophages than in adipocyte cells (Figure 4C). In addition, the effect of irisin was stronger on macrophage cells than on adipocyte cells.

## 4. Discussion

Adipose tissue (AT) is an endocrine organ producing factors that affect multiple structures in our bodies. In obese individuals, adipose tissue becomes a source of factors underlying the development of systemic pathologies such as insulin resistance, type 2 diabetes, cardiovascular diseases, cancers, and others (for review, see Reference [30]). Numerous studies have also indicated that the development of the above complications is related to the development of chronic inflammation associated with obesity, the main source of which is a macrophage influx into adipose tissue [4,31]. According to Weisberg et al. [31], fatty tissue composition differs between lean and obese individuals: it contains up to 40% of macrophages in obese subjects, while in lean individuals, this is only 10%. Increased infiltration of blood-derived monocytes into adipose tissue seems to be a key factor in the development of obesity-associated complications. In lean subjects, there is a prevalence of anti-inflammatory M2 macrophages, which coexist with Treg lymphocytes and eosinophil to create an anti-inflammatory environment [32]. On the other hand, adipose tissue macrophages in obese individuals present the proinflammatory M1 phenotype, with lower levels of Treg and eosinophil cells [33]. The research presented in this paper clearly indicates that both the glycosylated and non-glycosylated forms of irisin inhibited the inflammatory activation of adipocyte–macrophage cocultures. This was manifested by a reduction in both the expression and the release of proinflammatory cytokines, which are key in the development of obesity-related complications, such as TNF-α, IL-1β, and IL-6 (Figure 2). The main source of these factors, given the level of mRNA expression, was macrophages. In both cases, the action of nG-IR was most prominent and effective only at the 50-nM concentration. Monocyte influx into adipose tissue is stimulated by the release by adipocytes of chemotactic cytokine MCP-1 [34], while IL-1β released from adipose tissue macrophages plays a pivotal role in bone marrow macrophage recruitment [35]. The current study showed that irisin, by affecting the reduction of the expression and release of both MCP-1 and IL-1β, also indirectly reduced the potential for recruitment of macrophages to the adipose tissue environment. Both forms of irisin showed similar effects in this regard; however, analysis of the macrophage migration to supernatants from the culture showed that actual inhibition of the migration was observed only when the culture was incubated with nG-IR. The G-IR form was, in this case, ineffective, although a substantial but statistically insignificant decrease in the value of the index was noted. This result may suggest a potentially higher efficacy of nG-IR in relation to G-IR in inhibiting the macrophage influx into adipose tissue. It should also be noted that adipose tissue can affect distant structures via extracellular microvesicles, especially exosome-like vesicles (ELVs), which contain both proteins, mRNAs, and miRNAs (for review, see Reference [36]). As presented by Deng et al. [37], administration to lean mice of exosomes produced by adipocytes taken from obese individuals resulted in the activation of peripheral blood monocytes and increased release of proinflammatory TNF-α and IL-6, simultaneously leading to insulin resistance development in lean individuals. Considering the above-mentioned effects of irisin action, it cannot be excluded that irisin also has an impact on the quantity and quality of fat-released ELVs (research in progress). Nevertheless, we observed that myocyte C2C12 cells cultured with the addition of an adipocytic–macrophage coculture medium showed an improvement in glucose uptake when treated with medium taken from the nG-IR groups. Glucose uptake after using additional insulin stimulation was also improved when compared to the group with the addition of the conditioned medium from the groups without irisin. This effect was not so pronounced when only the addition of irisin was used. Similarly, in this case, the effect of nG-IR was stronger than that of G-IR (data not shown).

When analyzing the impact of irisin on adipocyte–macrophage interaction and obesity-related inflammation, proinflammatory alarmin HMGB1 should also be taken into account, whose elevated level has been noted in obese individuals [38]. The HMGB1 level is twice as high in adipose tissue from obese individuals as it is in lean subjects [39], and its expression rapidly increases after adipocyte differentiation [40]. Moreover, as presented by Guzmán-Ruiz et al. [41], HMGB1 in adipose tissue and 3T3 L1 adipocytes is abundantly deposited in the cytoplasm and not in the nucleus, as in the case of, for example, quiescent cells [42], and plays a pivotal role in the polarization of adipose tissue macrophages to M1 phenotypes [43]. The activation of AT by HMGB1 leads to excessive release of crucial proinflammatory cytokines such as TNF-α, IL-1β, or IL-6, which is related to the activation of the TLR4 pathway by HMGB1 [44] (for review, see Reference [45]). The current study demonstrated that irisin effectively decreased HMGB1 release by cocultures; however, nG-IR was much more effective in this respect than the G-IR form was. Our previous study, which was conducted on an individual culture of macrophages, showed that nG-IR effectively inhibited the TLR4/MyD88 pathway, leading to a reduction in the release of proinflammatory cytokines. This may be an additional explanation for the observed anti-inflammatory action of irisin. However, as shown in the present study, the effect of nG-IR was stronger in this respect than G-IR.

Moreover, this study also evaluated the effect of irisin on the production and release of key adipokines, such as leptin and adiponectin. Leptin is an adipokine that acts on the hypothalamus, exerting a significant influence on both food intake and the expenditure of energy. Under normal conditions, its elevated level reduces food intake and increases energy expenditure. A decrease in leptin level induces the opposite effect, i.e., an increase in food intake and a decrease in energy expenditure [30]. It needs to be highlighted that, in obese individuals, the leptin level is pathologically increased, and a state of leptin resistance is observed [46]. Moreover, studies have shown that leptin is an immunomodulating agent that affects both innate and acquired immunity (for review, see Reference [47]). Leptin increases the proliferation and activation of macrophages [48], which indirectly leads to an increase in proinflammatory cytokine release [49]. In our studies, we noted a decrease in leptin level due to incubation with both nG-IR and G-IR, which may explain, at least in part, the lack of an increase in macrophage proliferation, which was previously observed in individual macrophage cultures under nG-IR treatment [7]. In addition, by reducing the release of leptin, irisin reduces the proinflammatory activation of the entire system and thus reduces the risk of the development of insulin resistance. Research has shown that, in this respect, the effects of nG-IR and G-IR are equal. Another important adipokine, adiponectin, is known for its anti-inflammatory effect as well as for increasing the insulin sensitivity of liver cells, thereby leading to a decrease in hepatic glucose production [30]. Adiponectin levels in obese individuals significantly decrease, which, in combination with the observed increase in leptin level and activation of proinflammatory pathways, contributes to the development of insulin resistance and consequently also to type 2 diabetes [50,51]. Current research has clearly indicated that irisin eliminates the above-mentioned negative effect of obesity, leading to a decrease in the level of proinflammatory leptin, with an increase in the level of anti-inflammatory adiponectin. This effect was observed after the incubation of coculture cells in both the recombinant, non-glycosylated form of irisin as well as in its glycosylated form, which may suggest the possible therapeutic potential of irisin. An increased level of adiponectin was also observed in [52], who showed that irisin improves the function of perivascular adipose tissue by activating the heme oxygenase (HO)-1/adiponectin pathway. However, further research is necessary to verify the exact mechanisms of its action and its therapeutic potential.

## 5. Conclusions

Overall, our study showed that irisin, regardless of the presence or absence of its glycosylation, effectively inhibited the proinflammatory activation of adipocyte–macrophage cocultures. This may be the basis for a protective mechanism underlying a reduced risk of insulin resistance and type 2 diabetes development in obese patients. This study indicates that the therapeutic potential of irisin is not limited to its synthetic recombinant form. This prompts us to undertake research on the development of physical exercise programs that may have potentially therapeutic effects mediated by the proper level of irisin in our body. However, further studies are necessary to enable a detailed explanation of this phenomenon.
